# New Simple and Robust Method for Determination of Polarity of Deep Eutectic Solvents (DESs) by Means of Contact Angle Measurement

**DOI:** 10.3390/molecules27134198

**Published:** 2022-06-29

**Authors:** Łukasz Cichocki, Dorota Warmińska, Justyna Łuczak, Andrzej Przyjazny, Grzegorz Boczkaj

**Affiliations:** 1 Department of Process Engineering and Chemical Technology, Faculty of Chemistry, Gdansk University of Technology, G. Narutowicza St. 11/12, 80-233 Gdansk, Poland; s160191@student.pg.edu.pl (Ł.C.); justyna.luczak@pg.edu.pl (J.Ł.); 2 Department of Physical Chemistry, Faculty of Chemistry, Gdansk University of Technology, G. Narutowicza St. 11/12, 80-233 Gdansk, Poland; dorota.warminska@pg.edu.pl; 3 Advanced Materials Center, Gdansk University of Technology, G. Narutowicza St. 11/12, 80-233 Gdansk, Poland; 4 Department of Natural Sciences, Kettering University, 1700 University Avenue, Flint, MI 48504, USA; aprzyjaz@kettering.edu; 5 Department of Sanitary Engineering, Faculty of Civil and Environmental Engineering, Gdansk University of Technology, G. Narutowicza St. 11/12, 80-233 Gdansk, Poland

**Keywords:** DESs, polarity, hydrophobicity, contact angle, solvatochromism, materials characterization

## Abstract

The paper presents a new method for evaluating the polarity and hydrophobicity of deep eutectic solvents (DESs) based on the measurement of the DES contact angle on glass. DESs consisting of benzoic acid derivatives and quaternary ammonium chlorides–tetrabutylammonium chloride (TBAC) and benzyldimethylhexadecylammonium chloride (16-BAC)—in selected molar ratios were chosen for the study. To investigate the DESs polarity, an optical goniometer and an $E_{T}\left(30\right)$ solvatochromic scale based on Reichardt’s dye were used. The research demonstrated the high accuracy and precision of the developed procedure. The simplicity of the examination and the availability of basic equipment allow for the implementation of the developed method in routine investigations of DESs.

## 1. Introduction

Increasingly more research on DESs is carried out throughout the world, and their properties are being improved for effective application in science and industry: absorption [1], gas chromatography [2], and extraction [3]. The first step to understanding the nature of DESs is to study their physicochemical properties [4,5]. Some physicochemical properties, such as density or viscosity, can be easily measured. However, some of them are difficult to measure and, sometimes, almost impossible to determine for DESs. Such properties include polarity and hydrophobicity of DESs. For example, the study of the octanol-water partition coefficient [6] requires contact of DESs with water, which, as a strong solvating agent, displaces DES intramolecular hydrogen bonds with bonds between DES substrates and water molecules [7]. The aqueous decomposition of DESs to the starting materials means that it is not DESs that are actually investigated but the aqueous mixture or solution of the reactants forming DESs. For polarity measurement, reversed-phase high-performance liquid chromatography can be used, but it also requires converting DESs into solution in a given solvent [8]. Although solvents other than water can be used, they can also perturb the DES structure. Even nonpolar solvents can negatively affect the accuracy of the measurement due to the fact that practically the properties of two-component DESs in a solution can be treated as three-component DESs in terms of the physicochemical properties investigated. It follows from the fact that solvents such as methanol, which also can act as HBD, can form hydrogen bonds with the HBA component of the DESs, while acetone can present the opposite behavior as it can act as HBA. Such an effect was confirmed in the literature (DES—malonic acid–choline chloride–propanediol) [9]. Therefore, in this study, a method of determining DES polarity and hydrophobicity was sought without the need to prepare solutions, i.e., without interfering with the internal structure of DESs. The structure of DESs, as a mixture of at least two components having hydrogen bond donors (HBD) and hydrogen bond acceptors (HBA), is determined by the hydrogen bonds formed between HBD and HBA. The system of hydrogen bonds in the DES molecule is sensitive to changes related to the presence of an additional component, such as water or another solvent.

The contact angle between liquid drop and surface depends on their affinity. In the case of a surface having defined polarity, the contact angle is affected by the polarity or hydrophobicity of the liquid. On the other hand, in the case of the solvatochromic method, the interaction between the studied liquid and dye molecules results in an observed band shift. From this point of view, it could be expected that both phenomena could be correlated. In the case of strong correlation, it seems that contact angle measurement could be used in specific scenarios as a good alternative for the solvatochromic method. According to this hypothesis, this paper presents an attempt to use such a possibility for the characterization of DES properties using a simpler—contact angle-based—method. The contact angle is measured for DESs by the direct method of depositing a drop of DES on the reference surface. The method does not require the use of any solvents, only an optical goniometer and a reference surface. The method of measurement of contact angle is simple and provides direct results in a short time. In the present study, a second solvent-free method based on solvatochromism using Reichardt’s dye was also performed to measure the polarity for comparison purposes [10]. The solvatochromic responses of UV-vis absorption probes have been so far applied by Florindo et al. [11] for DESs based on choline chloride, DL-menthol, and TBAC and by Pandey et al. [12] for DESs containing ChCl and glycerol, urea, malonic acid, and ethylene glycol. Pandey et al. identified that the high polarity of the studied DESs was significantly influenced by HBD nature. Among the above four combinations, ChCl–Gly exhibited the highest $E_{T}\left(30\right)\;$ scale value. This observation was consistent with the results obtained by Florindo et al., who found that the polarity of DESs changes in the order: choline chloride–malonic acid > choline chloride–glycolic acid > choline chloride–levulinic acid. Solvatochromic indices to examine the polarities of DESs were also used by Abbott et al., who determined the polarities of choline chloride–glycerol DESs of different molar ratios, revealing a linear polarity increase with increasing ChCl concentration. In summary, the higher the number of carboxyl, hydroxyl, or carbonyl groups is in HBD, the higher polarity of the DESs composed of such HBD. This relationship results from the structure of the carboxyl group, which as a combination of the -C=O (carbonyl group) and the -OH (hydroxyl group), is more polar than the sole hydroxyl group or the carbonyl group.

Herein, for polarity measurements, DESs consisting of benzoic acid derivatives and quaternary ammonium chlorides–tetrabutylammonium chloride (TBAC) and benzyldimethylhexadecylammonium chloride (16-BAC)—in selected mole ratios were chosen. Selected DESs differed in polarity due to different structures of HBAs (TBAC containing short alkyl chains, 16-BAC with hydrophobic n-hexadecyl alkyl chain) but from the same class of chemical compounds—ammonium salts. For method calibration, water and rapeseed oil were used, while choline chloride–urea (1:2) as hydrophilic DESs and cineole-menthol (1:1) as hydrophobic DESs were used for data comparison. Thus, overall, both hydrophilic and hydrophobic DESs were used to present the applicability of the developed method for general DES characterization purposes.

## 2. Results and Discussion

The measurement of the polarity of DESs using the contact angle technique is an alternative to tests requiring the preparation of DES solutions in solvents. The high viscosity and low stability of DESs make working with DESs difficult and may require optimization of test conditions in order to obtain correct results. Polarity studies with the use of solvents eliminate the problem of recrystallization and high viscosity of DESs, but they completely disrupt the internal structure of DESs, which significantly changes the physicochemical properties of DESs. The contact angle measurement carried out in an optical goniometer allows the determination of contact angles of neat DESs. Measurement of the contact angle while meeting the requirements of the procedure provides the contact angles of DESs relative to the tested surface–glass. The contact angle, therefore, indirectly determines the affinity of DES molecules to the surface of the reference material. Two reference substances with extremely different properties were used in the study—water as a representative of a highly hydrophilic liquid and rapeseed oil as a representative of a highly hydrophobic liquid. The contact angle test itself requires obtaining a DES drop of the smallest possible diameter. A drop diameter as small as possible is very important in preventing the DES drop from spreading out. The spreading out of DES drops and other factors leading to the asymmetry of the drops distort the result of the test.

The contact angle should be identical, measured along the entire circumference of the drop’s contact with the surface. In the case of a goniometric measurement, the drop is projected onto a plane, and therefore, only two contact angles on the opposite sides of the drop can be measured. The final results of the contact angle determination were analyzed in terms of the similarity of the behavior of a given DES on glass with respect to water and rapeseed oil as reference substances. It is worth mentioning that DESs were prepared from dried components in closed vials. The contact of DESs with air during preparation was minimized. Both used measurement procedures were relatively quick; thus, the effect of humidity of air on the results was minimized. Simplicity and robustness of contact angle measurement are one of the big advantages of this method with respect to DES characterization. The results of contact angle measurements are summarized in Table 1.

**Figure 1:**
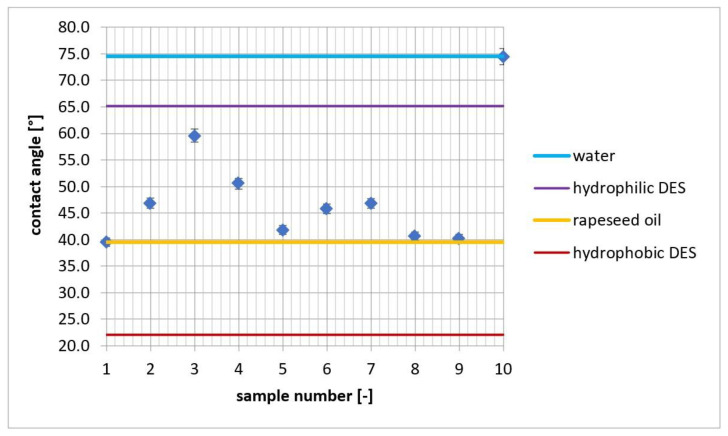
Contact angle: 1—rapeseed oil, 2–9—DESs, and 10—water—on glass.

DESs for which the contact angle values on glass were closest to water were classified as DESs with predominantly hydrophilic properties (DES 3), while DESs for which the contact angle values on glass were close to oil on glass were classified as hydrophobic (DESs 5, 8, and 9). DESs with contact angles intermediate between water and oil were classified as intermediate between hydrophilic and hydrophobic (DESs 2, 4, 6, and 7). The results are presented in Figure 1. Additionally, for comparison purposes, hydrophilic and hydrophobic DESs were used—choline chloride–urea (1:2) (measured contact angle on glass 65.2 ± 4.3 [°]) and cineole–menthol (1:1) (measured contact angle on glass 22.1 ± 3.6 [°] [13]), respectively. These values are in agreement with data presented in Table 1—hydrophobic DESs had on the glass surface a contact angle close to rapeseed oil, while hydrophilic DESs had values comparable with water.

Inspection of the graph reveals that DESs have different contact angle values depending on the molecular structure, which is reflected in the polarity and hydrophobicity of DESs. DES no. 3, consisting of TBAC and 3,5-dinitrobenzoic acid, was found to be the most hydrophilic. TBAC as a quaternary ammonium salt has four lone pairs of electrons on the chloride anion, which is a potential HBA, and the 3,5-dinitrobenzoic acid bonded to TBAC has two nitro groups that strongly deactivate the benzene ring, increasing the effect of the HBA–HBD interaction. The study also revealed the two most hydrophobic DESs—nos. 8 and 9. The highly hydrophobic nature of these DESs is mainly due to the n-hexadecyl groups from 16-BAC, showing much stronger hydrophobicity than the n-butyl groups of TBAC.

**Figure 2:**
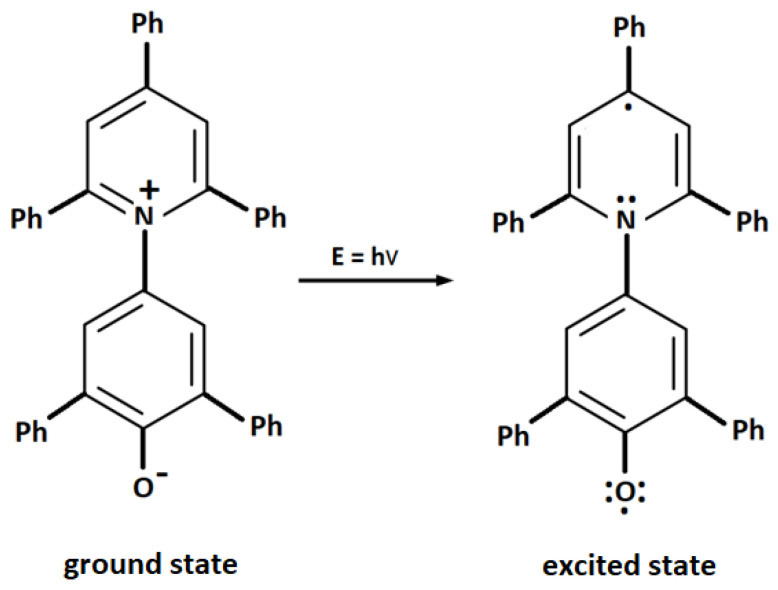
Reichardt’s betaine (own drawing based on [14]).

For comparative purposes, the polarity measurement was carried out using the second solvent-free method, based on solvatochromism, i.e., changes in absorption of electromagnetic radiation by dyes depending on the solvent in which they are dissolved. One of the largest solvatochromic effects (highest shift of absorption bands) is observed for Reichardt’s dye. Solvatochromism is another method of measurement of DES polarity without the need to prepare DES solutions, thus without affecting the internal structure of DESs. The structure of DESs and the DES–dye interactions affect the structural changes and charge distribution in the Reichardt’s dye molecule. As a result, the dye has different absorption of radiation at a particular wavelength depending on the environment in which it is present. Such a relationship is caused by shifts in charge transfer (CT) bands in the dye. The shifts of the CT bands result from the effect of DESs on the position of conjugated double bonds in the dye molecule. The excited state of the dye is stabilized by polar solvents through the phenomenon of solvation of the dye molecule by solvent molecules, hence the predominance of this form of betaine in the polar environment (Figure 2).

**Figure 3:**
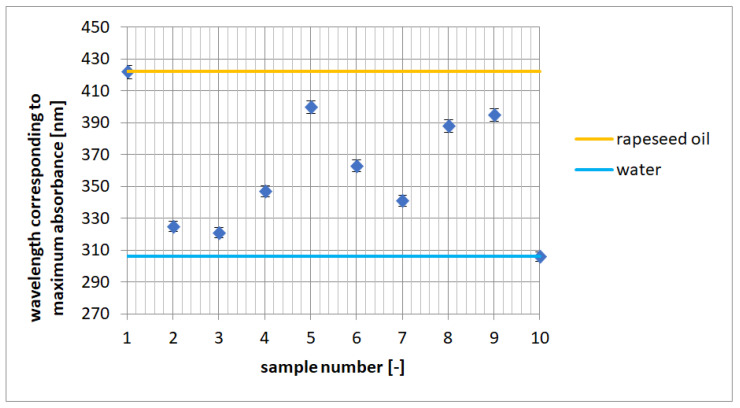
Wavelength corresponding to maximum absorbance for Reichardt’s dye: 1—rapeseed oil, 2–9—DESs, and 10—water.

The wavelengths corresponding to the maximum absorbance of the Reichardt’s dye in DESs relative to the reference substances—water and rapeseed oil reveal the shift in the absorption band of the dye. The comparison of the shifts in absorption maxima allows us to determine whether a given DES affects the dye in the same way as water (polar substance) or rapeseed oil (nonpolar substance)—Figure 3.

Measurement of the CT band shifts in the Reichardt’s dye became the basis for the creation of a polarity scale called $\;E_{T}\left(30\right)$. $E_{T}\left(30\right)$ values are based on measurements of the wavelength corresponding to the highest absorbance (Equation (1)). In addition to $E_{T}\left(30\right)$, the so-called normalized value $E_{T}^{N}\left(30\right)$ was introduced, in which the least polar substance was tetramethylsilane (TMS): $E_{T}^{N}\left(30\right)=0,$ and the most polar substance was water: $E_{T}^{N}\left(30\right)=1$. Hence, the normalized scale ranges from 0.000 for TMS to 1.000 for water. In this work, for the calculation of the normalized polarity scale, rapeseed oil was used as the least polar substance: $E_{T}^{N}\left(30\right)=0,\;$ and water was used as the most polar substance: $E_{T}^{N}\left(30\right)=1$.

(1) $$E_{T}\left(30\right)\left[\mathrm{kcal}/\mathrm{mol}\right]=hcN_{A}\nu_{max}\left({\mathrm{cm}}^{-1}\right)=\frac{28,591}{\lambda_{max}\left[\mathrm{nm}\right]}$$

(2) $$E_{T}^{N}\left(30\right)=\frac{E_{T}\left(30\right)-E_{T}\left(rapeseed\;oil\right)}{E_{T}\left(H_{2}O\right)-E_{T}\left(rapeseed\;oil\right)}\;$$

where *h*—Planck constant [Js], *c*—speed of light [m/s], *ν*—frequency [1/s], and *λ*—wavelength [nm].

On the basis of the wavelength corresponding to maximum absorbance and Equations (1) and (2) [14], the normalized values of the $E_{T}^{N}\left(30\right)$ polarity scale were calculated (Table 2).

Based on Table 2, it is possible to assess the similarity of a given DES to water, which demonstrates the hydrophilic nature of DES, or to rapeseed oil, which reveals the hydrophobic nature of DES. Values of $E_{T}^{N}\left(30\right)$ ranging from 0 to 0.25 were obtained for DES number 5, 8, and 9, which proves the hydrophobic nature of these DESs. Intermediate values of the polarity coefficient, ranging from 0.25 to 0.8, were obtained for DESs no. 2, 4, 6, and 7, while the highest similarity to water and the lowest to rapeseed oil was obtained for DES no. 3: $E_{T}^{N}\left(30\right)$ was in the 0.8–1 range.

Inspection of Table 3 reveals that both methods of DESs classification in terms of polarity are compatible. A Pearson correlation factor calculated for *E_T_* (30) and contact angle values was 0.834 confirming high correlation between obtained series of data. The two test methods (goniometric and solvatochromic) are independent and based on different DESs parameters, but ultimately, they indicate the same property of DESs—polarity. In the case of goniometric measurement of contact angle, the results obtained were based on the interactions between DESs and the reference surface. The contact angle indicates the affinity of DESs for a particular structural group constituting the reference material (glass–oxygen groups of silicon oxide). For the solvatochromic measurement, shifts in charge transfer (CT) bands in the Reichardt’s dye due to the presence of DES are used. Both parameters indirectly indicate DESs polarity and are related to its hydrophilicity but are based on completely different phenomena. Obtaining similar results using such different methods demonstrates the correctness of the determination. It is also clear that both methods of polarity assessment are universal and especially useful for testing complex (multicomponent) mixtures, in which the preparation of aqueous solutions is either impossible due to the low solubility of DESs or due to hydrolysis of DESs substrates in aqueous solutions.

The methods of determination of polarity described in this paper, especially the contact angle technique, can be widely used in the study of very complex mixtures. The goniometric method also has a great advantage over the solvatochromic procedure due to the simplicity of the procedure and the possibility of investigation of opaque samples, as well as the low cost of the test and its short duration. In many cases, the measurement can be simplified by taking a picture with a camera and image processing with appropriate graphics software. The only limitations of the goniometric method are substances whose very high viscosity and density prevent the free formation and detachment of drops from the goniometer needle.

## 3. Materials and Methods

### 3.1. Materials

The following reagents were used for the preparation of DESs: as HBA: TBAC and 16-BAC with purity greater than 97%—(Sigma Aldrich, Buchs, Switzerland) and as HBD acids: salicylic, 3,5-dinitrobenzoic, 3,5-dinitrosalicylic, 4-chlorosalicylic, acetylsalicylic, 4-tert-butylbenzoic, 4-methylbenzoic, and 5-sulfosalicylic with purity greater than 98%—(Sigma Aldrich, Buchs, Switzerland). Glass was used as the reference material for contact angle measurement.

### 3.2. Methods

#### 3.2.1. Preparations of DESs

The preparation of DESs was carried out in previously selected mole ratios, depending upon DES: HBA–HBD 1:1 and 3:2. In order to prepare DES, proper amounts of the reactants were weighed on a model AS.310.R2 analytical balance (RADWAG, Radom, Poland) with an accuracy of 0.1 mg so that the total mass of HBA and HBD ranged from 2 to 3 g. Next, the weighed amounts of the reactants were placed into a 5 mL screw cap vial along with a magnetic stir bar. The vials were capped and placed in a heating mantle using water as a heating medium and equipped with a magnetic stirrer (set model: 06 MSH PRO T, CHEMLAND, Stargard, Poland). Reactant mixtures were left for 120 min at 340–350 K with stirring at 1400–1500 rpm. After 120 min, all of the synthesized DESs were clear liquids. The molar compositions of the examined DESs are listed in Table 4.

#### 3.2.2. Polarity Measurement

##### Contact Angle

**Figure 4:**
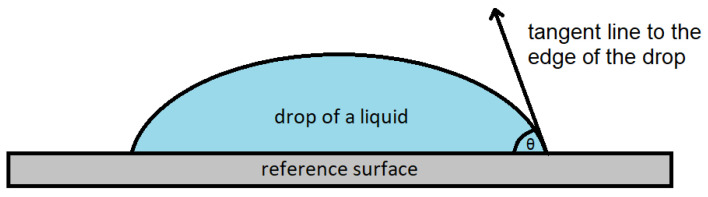
Graphical representation of contact angle determination using the sessile drop technique (θ—the contact angle). Own drawing based on [16].

The contact angle was measured with a model OCA 25 optical goniometer (DataPhysics, Filderstadt, Germany) using the sessile drop technique (Figure 4). Silicate glass, which is considered a material with a predominance of hydrophilic properties (oxygen groups of silicon (IV) oxide in the glass structure), was used as the reference surface. The glass surface was prepared by thoroughly cleaning it with methanol and leveling. Prior to sampling, DESs were heated and then brought to room temperature in order to ensure the representativeness of the sample. The samples were collected using a 1 mL plastic syringe. Based on the publication [15], a needle diameter of 0.55 mm was selected as the optimum to generate DES drops on the tested surface. The same needle diameter was used for all samples to facilitate the comparison of the obtained results. DESs drops were produced by the syringe needle placed perpendicularly to the tested surface. The tip of the needle was 30 mm above the test surface. The movement of the syringe plunger was controlled by the stepper motor, which ensured a slow increase in the droplet at the tip of the needle until the drop was detached by gravity.

The results from the goniometer software were recorded after obtaining identical values of both contact angles on both sides of the drop. Each measurement was carried out on glass at room temperature in triplicate. Contact angle values were computed by goniometer software using the Young–Laplace fit (Equation (1)). This model uses the DES drop shape through the radius of the drop projection onto the plane. The knowledge of the Laplace pressure (Δ*P*) by the software allows the calculation of the contact angle on the surface

(3) $$\Delta P=\gamma \left(\frac{1}{R_{1}}-\frac{1}{R_{2}}\right)\;\;$$

where *γ*—surface tension [N/m], Δ*P*—Laplace pressure [Pa], and *R*—radius [m] [16]. 

##### Solvatochromism

This method requires a solution of Reichardt’s dye in DES at a concentration of approximately 0.3 mg/mL of sample. The dye concentration in DESs was optimized so that the absorbance as a function of dye concentration ranged from 0 to 1 (linear range of the Lambert–Beer law). A model DR 5000 spectrophotometer (Hach Lange, Ames, IA, USA) was used in the experiments. Neat DESs were used as a reference in spectrophotometric analysis in order to eliminate the effect of background on UV-VIS absorbance measurements. Next, the absorption spectra of the dye solutions in DESs were taken, and the wavelength of maximum absorbance was determined.

## 4. Conclusions

The results obtained in this study revealed that the goniometric (contact angle measurement) method of testing the polarity of DESs on glass can be used to determine the hydrophilicity/hydrophobicity of DESs with respect to each other or with respect to reference substances. This method has a number of advantages over the methods using solvents. Firstly, it does not require the use of solvents, most of which are toxic and require proper disposal after use. Secondly, the goniometric method is consistent with the principles of green chemistry, and it is completely emission-free. The study also demonstrated the compatibility of the goniometric method with the solvatochromic method, which requires expensive and difficult to synthesize dyes. A very small amount of sample is needed for a goniometric test, and the entire sample can be recovered from the reference surface. In the case of other polarity testing methods, also the solvatochromic method, the preparation of dye solutions in DESs is required, which contaminates the DES samples and precludes their subsequent use. Thus, the goniometric method is currently the most ecological and least expensive method of polarity testing, and there are also opportunities for its further development and optimization.

## Figures and Tables

**Table 1:** Contact angle (CA) values of DESs on glass.

Sample	HBA	HBD	Mole Ratio	CA Glass
1—Hydrophobic Liquid Ref.	Rapeseed oil			39.5
Hydrophobic DES Ref.	Cineole	Menthol	1:1	22.1
2	TBAC	Salicylic acid	3:2	46.9
3	TBAC	3,5-dinitrobenzoic acid	1:1	59.6
4	TBAC	3,5-dinitrosalicylic acid	1:1	50.6
5	TBAC	4-chlorosalicylic acid	1:1	41.8
6	TBAC	acetylsalicylic acid	3:2	45.8
7	TBAC	4-tert-butylbenzoic acid	1:1	46.8
8	16-BAC	Salicylic acid	3:2	40.6
9	16-BAC	Acetylsalicylic acid	3:2	40.2
Hydrophilic DES Ref.	Choline chloride	Urea	1:2	65.2
10—Hydrophilic Liquid Ref.	Water			74.5

**Table 2:** Calculated normalized values for $E_{T}^{N}\left(30\right)$ polarity scale.

Sample No. *	λ_max_ [nm]	$E_{T}\left(30\right)[\mathrm{kcal}/\mathrm{mol}]$	$E_{T}^{N}\left(30\right)$ [-]
1 (water)	306	93.4	1.00
2	325	88.0	0.79
3	321	89.1	0.83
4	347	82.4	0.57
5	400	71.5	0.15
6	363	78.8	0.43
7	341	83.8	0.63
8	388	73.7	0.23
9	395	72.4	0.18
10 (rapeseed oil)	422	67.8	0.00

* Sample No. 1—water, 2–9—DESs, and 10—rapeseed oil.

**Table 3:** Classification of DESs samples in terms of polarity.

Scale	Polarity/Hydrophilicity		
	High	Intermediate	Low
	DESs Sample No.		
Goniometric	3	2, 4, 6, 7	5, 8, 9
Solvatochromic	3	2, 4, 6, 7	5, 8, 9

**Table 4:** Mole ratios of investigated DESs.

HBA	HBD	Molar Ratio
TBAC	Salicylic acid	3:2
TBAC	3,5-dinitrobenzoic acid	1:1
TBAC	3,5-dinitrosalicylic acid	1:1
TBAC	4-chlorosalicylic acid	1:1
TBAC	Acetylsalicylic acid	3:2
TBAC	4-tert-butylbenzoic acid	1:1
16-BAC	Salicylic acid	3:2
16-BAC	Acetylsalicylic acid	3:2

## Sample Availability

Samples of the compounds used for DESs formation are available from the authors.

## References

[1] García G., Aparicio S., Ullah R., Atilhan M. (2015). Deep Eutectic Solvents: Physicochemical Properties and Gas Separation Applications. Energy Fuels.

[2] Momotko M., Łuczak J., Przyjazny A., Boczkaj G. (2021). First deep eutectic solvent-based (DES) stationary phase for gas chromatography and future perspectives for DES application in separation techniques. J. Chromatogr. A.

[3] Tang B., Zhang H., Row K.H. (2015). Application of deep eutectic solvents in the extraction and separation of target compounds from various samples. J. Sep. Sci..

[4] Zhu J., Yu K., Zhu Y., Zhu R., Ye F., Song N., Xu Y. (2017). Physicochemical properties of deep eutectic solvents formed by choline chloride and phenolic compounds at T = (293.15 to 333.15) K: The influence of electronic effect of substitution group. J. Mol. Liq..

[5] Jafari K., Fatemi M.H., Estellé P. (2021). A short overview of the thermophysical properties and current use as base fluid for heat transfer nanofluids. J. Mol. Liq..

[6] Farias F.O., Passos H., Lima A., Mafra M., Coutinho J.A.P. (2017). Is It Possible to Create Ternary-like Aqueous Biphasic Systems with Deep Eutectic Solvents?. ACS Sustain. Chem. Eng..

[7] Ma C., Laaksonen A., Liu C., Lu X., Ji X. (2018). The peculiar effect of water on ionic liquids and deep eutectic solvents. Chem. Soc. Rev..

[8] Du C.M., Valko K., Bevan C., Reynolds D., Abraham M.H. (2001). Rapid method for estimating octanol-water partition coefficient (LOG POCT) from isocratic rp-hplc and a hydrogen bond acidity term (A). J. Liq. Chromatogr. Relat. Technol..

[9] Jablonsky M., Majova V., Ondrigova K., Sima J. (2019). Preparation and characterization of physicochemical properties and application of novel ternary deep eutectic solvents. Cellulose.

[10] Abbott A.P., Harris R.C., Ryder K.S., D’Agostino C., Gladden L.F., Mantle M.D. (2011). Glycerol eutectics as sustainable solvent systems. Green Chem..

[11] Florindo C., McIntosh A.J.S., Welton T., Branco L.C., Marrucho I.M. (2017). A closer look into deep eutectic solvents: Exploring intermolecular interactions using solvatochromic probes. Phys. Chem. Chem. Phys..

[12] Pandey A., Pandey S. (2014). Solvatochromic probe behavior within choline chloride-based deep eutectic solvents: Effect of temperature and water. J. Phys. Chem. B.

[13] Zamora L., Benito C., Gutierrez A., Alcande R., Alomari N., Al Bodour A., Atilhan M. (2022). Nanostructuring and macroscopic behavior of type V deep eutectic solvents based on monoterpenoids. Phys. Chem. Chem. Phys..

[14] Reichardt C. (1994). Solvatochromic Dyes as Solvent Polarity Indicators. Chem. Rev..

[15] Vuckovac M., Latikka M., Liu K., Huhtamäki T., Ras R.H.A. (2019). Uncertainties in contact angle goniometry. Soft Matter.

[16] Song B., Ju J., Springer J. (1996). Determination of Interfacial Tension from the Profile of a Pendant Drop Using Computer-Aided Image Processing 2. Experimental. J. Colloid Interface Sci..

